# Right heart echocardiographic variables and prediction of clinical severity in dogs with pulmonary stenosis

**DOI:** 10.1111/jvim.16679

**Published:** 2023-04-10

**Authors:** Valentina Patata, Tommaso Vezzosi, Federica Marchesotti, Eric Zini, Rosalba Tognetti, Oriol Domenech

**Affiliations:** ^1^ Department of Veterinary Sciences University of Pisa Pisa Italy; ^2^ Clinic for Small Animal Internal Medicine, Vetsuisse Faculty University of Zurich Zurich Switzerland; ^3^ Department of Animal Medicine, Production and Health University of Padova Legnaro, PD Italy

**Keywords:** fractional area change of the, pulmonary valve area, right atrium, right ventricle, right ventricle, tricuspid plane systolic excursion, velocity time integral

## Abstract

**Background:**

Pulmonary stenosis (PS) usually is evaluated using echocardiography. A multiparametric approach, in addition to the maximum pressure gradient (PG), might be indicated to better characterize PS severity and address its management.

**Hypothesis/Objectives:**

Our hypothesis was that right heart size and function are associated with echocardiographic and clinical severity of pulmonary stenosis in dogs.

**Animals:**

Client‐owned dogs with PS.

**Methods:**

Prospective, multicenter, observational study. Enrolled dogs underwent complete echocardiographic examination. Associations among right heart echocardiographic variables, PS transvalvular PG >80 mm Hg and presence of clinical signs (exercise intolerance, syncope, right‐sided congestive failure, or some combination of these) were assessed using logistic regression analysis.

**Results:**

Eighty‐eight dogs with PS. Twenty‐eight dogs were symptomatic. Increased right ventricular end‐diastolic free wall thickness (odds ratio [OR] > 100; 95% confidence interval [95%CI], 50‐ > 100; *P* = .01) and decreased aorta‐to‐pulmonary artery velocity time integral ratio (OR, < 0.001; 95%CI, 0.0‐0.001; *P* = .005) were independently associated with PS PG >80 mm Hg. Decreased tricuspid annular plane systolic excursion (OR, 0.35; 95%CI, 0.15‐0.77; *P* = .01) and increased right ventricular end‐diastolic area (OR, 1.4; 95%CI, 1.08‐2.02; *P* = .01) were independently associated with clinical severity.

**Conclusion and Clinical Importance:**

Structural and functional right heart echocardiographic variables are associated with echocardiographic and clinical severity in dogs with PS. A multiparametric approach is advised to better assess PS severity.

**Abbreviations**
AV_a_
: aortic valve annulus
AV_a_:PV_a_
: aortic to pulmonary artery annulus ratio
BSA: body surface area
iPVA: pulmonary valve area index
iFAC: fractional area change of the right ventricle index
iRAA: right atrium area indexed to body surface area
iRVEDA: right ventricular area at end‐diastole indexed to body surface area
iRVFWd: right ventricular free wall thickness index
iTAPSE: tricuspid plane systolic excursion index
PG: pressure gradient
PS: pulmonary stenosis
PV_a_
: pulmonic valve annulus
PV_Max_
: transpulmonary peak velocity
R‐CHF: right congestive heart failure
RV: right ventricle
TR: tricuspid regurgitation
VTI_Ao_
: aortic flow velocity time integral
VTI_Ao_:VTI_PV_
: aorta to pulmonary artery velocity time integral ratio
VTI_PV_
: pulmonary flow velocity time integral
V_MaxAo_:V_MaxPV_
: aorta to pulmonary artery velocity ratio

## 1 INTRODUCTION

Pulmonary stenosis (PS) is a common congenital heart diseases in dogs.^1^, ^2^ Most dogs are asymptomatic at the time of diagnosis, but some can develop clinical signs such as exercise intolerance, syncope, and right‐sided congestive heart failure (R‐CHF). Previous studies indicated that the presence of clinical signs, young age at diagnosis, pulmonary annulus hypoplasia, pulmonary transvalvular pressure gradient (PG), and severity of tricuspid regurgitation (TR) adversely affect survival in dogs with PS.^3^, ^4^, ^5^ In addition to clinical signs, echocardiography is the most widely used method to evaluate PS severity. According to current guidelines, severity is based on the PG obtained by transpulmonary peak velocity (PV_Max_) using the modified Bernoulli equation.^6^ However, assessing PS severity based only on PG might be inaccurate, and a multiparametric approach is expected to add important information for assessment of PS severity, management, and possibly prognosis.^7^, ^8^ In fact, as demonstrated from Gorlin's formula, PG is an echocardiographic parameter that is highly influenced by cardiac output compared to other echocardiographic variables such as the pulmonary valve area index (iPVA), aortic (V_MaxAo_) to pulmonary maximum velocity (V_MaxPV_) ratio (V_MaxAo_:V_MaxPV_), and the aortic to pulmonary artery velocity time integral ratio (VTI_Ao_:VTI_PV_).^7^ Therefore, in some cases, PG might underestimate or overestimate the severity of PS, and thus a multiparametric approach might be indicated. Moreover, an echocardiographic multiparametric approach is recommended in human medicine to assess the severity of valve stenosis.^9^ In particular for aortic stenosis, it is suggested to use valve area and mean transvalvular PG in all patients, whereas in selected patients, when additional information is needed, the velocity ratio and planimetry of the anatomic valve area should be considered.^9^ For PS, it is suggested to evaluate associated lesions such as right ventricular (RV) hypertrophy and RV enlargement.^9^ Studies in human and veterinary medicine indicate that echocardiographic assessment of right heart size and function is important to predict the presence of clinical signs and define prognosis in patients with right heart disease, both acquired and congenital.^8^, ^9^, ^10^, ^11^, ^12^ However, a recent study evaluated right heart size and function in dogs affected by PS, and suggested the importance of a multiparametric approach for echocardiographic evaluation.^8^ Therefore, our aim was to verify the hypothesis that right heart size and function are associated with echocardiographic and clinical severity of PS in dogs.

## 2 MATERIALS AND METHODS

### 2.1 Animals

Ours was a prospective, multicenter, observational study that enrolled client‐owned dogs with PS at the Anicura Istituto Veterinario Novara and at the Department of Veterinary Sciences of the University of Pisa, with the owner giving signed consent. Dogs were consecutively included in the study over a 2.5‐year period if they had clinical and echocardiographic findings compatible with congenital PS. Exclusion criteria were presence of other cardiac (eg, patent foramen ovale, tricuspid dysplasia) or systemic diseases, presence of atrial fibrillation, and if sedation was needed to perform echocardiography. Presence or absence of patent foramen ovale was assessed based on color Doppler examination in all dogs, and in selected unclear cases a bubble study was performed. Treatment with atenolol was not considered an exclusion criterion. Dogs were divided into 2 groups of PS echocardiographic severity based on peak PG: PS with a maximum (max) PG ≤80 mm Hg, and PS with a max PG >80 mm Hg. Differences on right heart echocardiographic variables were evaluated between these 2 groups of PS severity. The association between right heart echocardiographic variables and echocardiographic severity (PS with max PG >80 mm Hg) was assessed using logistic regression analysis. To assess clinical severity dogs were divided into 2 groups: asymptomatic and symptomatic. Dogs were considered symptomatic if the owner reported exercise intolerance, syncope, R‐CHF, or a combination of these. Right‐sided congestive heart failure was defined as the presence of ascites associated with jugular venous distension and dilated caudal vena cava.

### 2.2 Echocardiographic examination

All dogs underwent standard transthoracic echocardiography, restrained in right and left lateral recumbency, with a simultaneous ECG tracing.^13^ Echocardiographic examinations were performed by a board‐certified cardiologist (OD) or a resident (VP, TV, FM) under the direct supervision of the same board‐certified cardiologist using Vivid iQ^e^ (GE Healthcare, 20 126, Milan, Italy) and Aplio 300 (Canon Medical Systems Europe, Zoetemermeer, the Netherlands) ultrasound systems with multi‐frequency phased‐array transducers. All measurement were performed by a single investigator (VP) and 3 cardiac cycles in sinus rhythm for each variable were averaged and used for statistical analysis. All valves were evaluated morphologically using 2‐dimensional (2D) imaging and interrogated using color Doppler echocardiography. The tricuspid valve apparatus was carefully evaluated in each dog to exclude the presence of tricuspid valve dysplasia, as previously described.^14^ Tricuspid dysplasia was considered when anomalies of the valve leaflets (eg, thickened, elongated, short, fused) or chordae tendineae (eg, short, long, absent) or papillary muscle (eg, long, short, fuse, direct attachment with the valve leaflet), or some combination of these was present.^14^ Aortic valve (AV_a_) and pulmonic valve (PV_a_) diameters were measured in early systole between the hinge points of maximally opened leaflets from the right parasternal long axis (RPLA) view optimized with the left ventricular outflow tract and from the right parasternal short axis (RPSA) view at the level of the heart base, respectively, in early to mid‐systole, and the AV_a_:PV_a_ ratio was calculated.^4^ The morphology of PS was evaluated and classified as type A, type B, and intermediate as previously described.^4^ Furthermore, the AV_a_:PV_a_ ratio was utilized to define PV_a_ hypoplasia when the AV_a_:PV_a_ > 1.2.^4^, ^6^ Briefly, PS type A was defined when there was fusion and thickening of the valve cusps associated with a normal PV_a_, whereas PS type B was considered when the dog had PV_a_ hypoplasia associated with thickened and immobile valve cusps.^6^ Pulmonary stenosis with intermediate morphology was defined when the valve had characteristics between type A and B PS.^4^ Moreover, the presence of supravalvular and subvalvular stenosis as well as the presence of coronary artery anomaly were evaluated as previously described.^5^, ^15^ Subvalvular and supravalvular PS were distinguished based on 2D anatomy of the right ventricular outflow tract, pulmonic valve and main pulmonary artery structures and evaluating the site of the narrowing and increased velocity seen with spectral and color Doppler. In particular, subvalvular PS was considered when the narrowing, generally determined by the presence of fibrous ring or fibromuscular hypertrophy, was below the pulmonic valve, whereas supravalvular PS was diagnosed when the narrowing was above the pulmonary valve, also including narrowing of the sino‐tubular junction.^5^, ^6^, ^15^, ^16^ Aortic flow was acquired from the subcostal view using pulsed wave Doppler, with the sample volume positioned between the hinge point of valve leaflets, and pulmonary flow was obtained from the right parasternal short axis view using continuous wave Doppler.^5^ Maximum transvalvular aortic velocity (Ao_Vmax_) and PV_Vmax_ was obtained and Ao_Vmax_:PV_Vmax_ ratio was calculated. Velocity time integrals of aortic flow (VTI_Ao_) and pulmonic flow (VTI_PA_) were obtained after the manual tracing of flow profiles and mean PG were generated by the machine's software. Velocity time integrals were used to calculate the VTI_Ao_:VTI_PV_ ratio and pulmonary valve area (PVA) using the continuity equation as follows: (cross sectional area of the aortic valve x VTI_Ao_)/VTI_PV_.^6^ Assuming that the aortic root is a circle, cross sectional area of the aortic valve was obtained using the formula: π x (AV_a_/2)^2^. The PVA then was indexed to each dog's body surface area (BSA) as previously described.^6^, ^7^

The left apical 4 chamber view optimized for the right heart was used to evaluate right heart size and function during echocardiographic measurements. In particular, right atrium area (RAA) was measured by planimetry at the end of ventricular systole from the lateral aspect of the tricuspid annulus to the septal aspect, excluding the area between the leaflets and annulus, following the right atrium endocardium and excluding the caudal vena cava.^16^ The RAA index (iRAA) was calculated by dividing RAA by BSA.^17^ Right ventricular end diastolic area (RVEDA) and end systolic area (RVESA) were measured by planimetry, tracing the lateral aspect of the tricuspid annulus to the septal aspect and excluding the area of the tricuspid annulus, trabecular structures and papillary muscles following the RV endocardium.^18^ The RVEDA index (iRVEDA) was calculated as the ratio of RVEDA to BSA.^18^

Right ventricular free wall thickness at end‐diastole (RVFWd) was measured at the mid‐ventricular level excluding the pericardium and including the endocardium, from the left apical 4‐chamber view as previously described.^19^ The RVFWd was indexed to body weight (BW; iRVFWd = cm/kg^0.250^).^19^

Right ventricular systolic function was assessed by measuring tricuspid annular plane systolic excursion (TAPSE) and fractional area change of the RV (FAC). The TAPSE was obtained from M‐mode recordings of the lateral aspect of the tricuspid valve annulus seen from the left apical 4‐chamber view optimized for the right heart.^20^, ^21^ The FAC was derived from the RVEDA and RVESA using the formula: FAC = [(RVEDA‐RV end systolic area)/RVEDA] x 100.^20^ Both TAPSE and FAC were indexed to BW using the previously published scaling exponent: iTAPSE in mm/kg^0.297^ and iFAC in %/kg^−0.097^.^20^ Tricuspid regurgitation severity was qualitatively evaluated using color Doppler and continuous wave Doppler.^13^, ^17^, ^22^ Mild TR was considered when a small (<25% of RAA), central jet signal on the color Doppler and a faint parabolic TR jet signal on the continuous wave Doppler were present. Moderate TR was considered when an intermediate TR jet (>25% and <50% of RAA) on the color Doppler and a dense parabolic TR jet signal on the continuous wave Doppler were observed. Severe TR was considered when a very large central jet or an eccentric jet (>50% of RAA) impinging on the wall on the color Doppler and a dense triangular with early peaking TR jet signal were seen on the continuous wave Doppler. For statistical purposes, the following scores were assigned to TR severity: no TR = 0, mild TR = 1, moderate TR = 2, severe TR = 3.

### 2.3 Statistical analysis

Statistical analysis was performed using commercially available statistical software (SAS system software, version 9.4‐SAS Institute Inc., Cary, NC, USA and MedCalc Statistical Software version 19.3.1‐MedCalc Software Ltd, Ostend, Belgium). A sample size calculation was performed using a paired test comparing 2 correlated means and specifying SDs of the differences. To calculate sample size, we used the results reported in a previous study^8^ comparing dogs with a PS PG ≤80 mm Hg and those with a PS PG >80 mm Hg. Considering the iPVA, VTI_Ao_:VTI_PV_ ratio and iTAPSE means and SDs, minimum sample sizes of 8, 7 and 11 subjects were calculated, respectively, to attain a power of 0.8 and an *α* of 0.05.

Normality of data was tested using the Shapiro‐Wilk test. Continuous data was reported as mean and SD if normally distributed or median and minimum and maximum if not normally distributed. Differences of the echocardiographic variables between dogs with transvalvular PG ≤80 mm Hg and > 80 mm Hg were evaluated using Mann‐Whitney or Student's t tests, depending on distribution of the data. Moreover, counting data were compared using a 2 proportions *z* test. Univariate logistic regression analysis was performed to identify echocardiographic variables associated with PS PG >80 mm Hg and with presence of clinical signs. Next, echocardiographic variables were entered into a multiple linear regression model based on the results of the univariate analysis (variables that yielded *P* < .05) and on the collinearity analysis. Collinearity was performed using the eigenvalues, condition indices, and decomposition of the variances of the estimates with respect to each eigenvalue.^23^ Multivariate logistic regression analysis was performed to identify echocardiographic variables independently associated with echocardiographic severity (PG >80 mm Hg) and clinical severity (if the owner reported exercise intolerance, syncope, R‐CHF, or a combination of these). Moreover, a preliminary analysis, including both univariate and multivariate logistic regression analysis, was done to evaluate which echocardiographic variables were associated with the presence of R‐CHF.

Receiver operating curve analysis and Youden index were used to identity the best echocardiographic cut‐offs of the variables independently associated with clinical severity and the presence of R‐CHF.

## 3 RESULTS

The study included 88 dogs with PS; 36 were females and 52 males with a median age of 0.8 years (minimum‐maximum, 0.2‐13 years) and a median BW of 10 kg (minimum‐maximum, 0.7‐17 kg). The sample included several different breeds: French Bulldog (n = 25), English Bulldog (n = 12), American Staffordshire Terrier (n = 4), Pinscher (n = 4), Boxers (n = 3), Yorkshire Terrier (n = 3), Cavalier King Charles Spaniel (n = 2), English Cocker Spaniel (n = 2), Golden Retriever (n = 2), Jack Russell Terrier (n = 2), Miniature Schnauzer (n = 2), Newfoundland (n = 2), and 1 each of American Bulldog, Belgian Shepherd, Bernese Mountain dog, Bloodhound, Continental Bulldog, Italian Bracco, Rhodesian ridgeback, Shetland Shepherd, Spitz, Whippet and 15 mixed‐breed dogs. The study included 60 dogs (68.1%) with type A PS, 14 (15.9%) with type B PS, 3 (3.5%) with intermediate type PS, and 11 dogs (12.5%) with valvular and supravalvular PS. Three dogs had a suspected coronary artery anomaly type R2A (3.8%), 1 of which was confirmed by ECG‐gated computed tomography. Based on the max PG, 29 dogs had PS PG ≤80 mm Hg and 59 had a PS PG >80 mm Hg. Twenty‐eight dogs were symptomatic: 14 dogs were presented with exercise intolerance, 8 with syncope, and 11 had R‐CHF. Four of the dogs with syncope also had exercise intolerance. Among dogs presented with R‐CHF, 2 had syncope and 4 had exercise intolerance. Twenty‐nine dogs (32.9%) were receiving atenolol (median dosage, 1 mg/kg/day; minimum‐maximum, 0.5‐2 mg/kg/day) at the time of examination.

Echocardiographic variables in dogs with PS are summarized in Table 1. Dogs with PS PG >80 mm Hg had significantly higher AV_a_;PV_a_ (*P* = .04), iRAA (*P* < .001), iRVEDA (*P* = .003) and iRVFWd (*P* < .001) and significantly lower V_MaxAo_:V_MaxPV_ (*P* < .001), VTI_Ao_:VTI_PV_ (*P* < .001), and iPVA (*P* < .001). The iTAPSE (*P* = .12) and iFAC (*P* = .25) were not significantly different between groups, however the percentage of dog having decreased iTAPSE was significantly lower in the PS PG ≤80 mm Hg group than in PS PG >80 mm Hg group. Tricuspid regurgitation was present in 55 of 88 (62.5%) dogs with PS. In particular, in dogs with a PS PG ≤80 mm Hg, 6 had mild TR (20.6%), 1 moderate TR (3.4%) and only 1 dog had severe TR (3.4%), which was affected by RV systolic dysfunction evaluated based on a marked decrease of iTAPSE and iFAC. Among dogs with PS PG >80 mm Hg, 28 had mild TR (47.4%), 12 moderate TR (20.3%), and 7 had severe TR (11.8%).

**TABLE 1:** Echocardiographic variables in 29 dogs with a pulmonary stenosis pressure gradient ≤80 mm Hg and 59 dogs with a pulmonary stenosis pressure gradient >80 mm Hg.

Variables	PS PG ≤ 80 mm Hg (n = 29)	PS PG > 80 mm Hg (n = 59)	*P*
Mean PG (mm Hg)	32.6 ± 11.8	81.9 ± 23.6	<.001
AV_a_:PV_a_	1.0 (0.85‐1.17)	1.1 (0.72‐2.3)	.04
V_MaxAo_:V_MaxPV_	0.36 (0.18‐0.76)	0.22 (0.12‐1)	<.001
VTI_Ao_:VTI_PV_	0.27 (0.13‐0.66)	0.13 (0.04‐0.29)	<.001
iPVA (cm^2^/m^2^)	0.70 (0.25‐2)	0.32 (0.24‐0.68)	<.001
iRAA	7.1 (5.7‐27.8)	12.5 (5.6‐46.3)	<.001
>10.2 cm^2^/m^2^	3/29 (10.3%)	33/59 (55.9%)	<.001
iRVEDA	7.5 (4.7‐20.6)	8.9 (4.7‐39.4)	.003
>11.6 cm^2^/m^2^	2/29 (6.8%)	15/59 (25.4%)	.04
iRVFWd	0.34 ± 0.07	0.60 ± 0.2	<.001
>0.39 cm/kg^0.250^	5/29 (17.2%)	51/59 (86.4%)	<.001
iTAPSE	5.9 ± 1.4	5.4 ± 1.1	.12
<4.8 mm/kg^0.297^	2/29 (6.8%)	20/59 (33.8%)	.01
iFAC	66.0 ± 13.0	61.7 ± 17.6	.25
<46.3%/kg^−0.097^	1/29 (3.4%)	9/59 (15.2%)	.10

*Note*: Data reported as mean ± SD or median (range). Moreover, for specific echocardiographic variables are reported the number and percentage (within brackets) of dogs which present the value above (iRAA, IRVAd, and iRVFWd) and under for (for iTAPSE and IFAC) the previously reported upper or lower reference limit respectively.^7^, ^17^, ^18^, ^19^

Abbreviations: AV_a_:PV_a_, aorta‐to‐pulmonary artery annulus ratio; iFAC, fractional area change indexed to body weight; iPVA, pulmonary valve area indexed to body surface area; iRAA, right atrial area indexed to body surface area; iRVAd, right ventricular area at end‐diastole indexed to body surface area; iRVFWd, right ventricular free wall thickness at end‐diastole indexed to body weight; iTAPSE, tricuspid annular plane systolic excursion indexed to body weight; Mean PG, mean transpulmonary pressure gradient; Max PG, maximum transpulmonary pressure gradient; PG, pressure gradient; PS, pulmonary stenosis; V_MaxAo_:V_MaxPV_, aorta to pulmonary artery maximum transvalvular velocity ratio; VTI_Ao_:VTI_PV_, aorta‐to‐pulmonary artery velocity time integral ratio.

Results of univariate and multivariate analysis to identify the echocardiographic variables associated with max PG > 80 mm Hg are presented in Table 2. Decreased VTI_Ao_:VTI_PV_ and increased iRVFWd were independently associated with PS max PG >80 mm Hg. In particular, for each increase of 1 of VTI_AV_/VTI_PV_ the risk of having a PS PG >80 mm Hg is decreased by >99%, whereas for each increase of 1 cm/kg^0.250^ of iRVFWd the risk of having a PS PG >80 mm Hg is increased by >100 times.

**TABLE 2:** Results of univariate and multivariate logistic regression analysis of the echocardiographic variables associated with echocardiographic severity of pulmonary stenosis (pressure gradient >80 mm Hg).

Variables	Univariable logistic regression		Multiple logistic regression	
	OR (95% CI)	*P* value	OR (95% CI)	*P* value
AV_a_:PV_a_	29.8 (1.5‐575)	.02		.90
Mean PG	1.34 (1.13‐1.5)	<.001		‐
V_MaxAo_:V_MaxPV_	0.001 (0.0‐0.010)	<.001		.57
VTI_Ao_:VTI_PV_	<0.01 (0.0‐0.01)	<.001	**<0.01 (0.0‐0.01)**	**.005**
iPVA	<0.010 (0.0‐0.013)	<.001		.78
iRAA	1.34 (1.1‐1.6)	.001		.70
iRVEDA	1.2 (1.01‐1.46)	.03		.79
iRVFWd	>100 (50‐>100)	<.001	**>100 (50‐>100)**	**.007**
iTAPSE	‐	.12		‐
iFAC	‐	.25		‐
TR severity	5.1 (2.2‐11.7)	<.001		.68

*Note*: The bold values represent the variables resulted statistically significant in the multivariable analysis.

Abbreviations: 95% CI, 95% confidence interval; AV_a_:PV_a_, aorta‐to‐pulmonary artery annulus ratio; iFAC, fractional area change indexed to body weight; iPVA, pulmonary valve area indexed to body surface area; iRAA, right atrial area indexed to body surface area; iRVEDA right ventricular area at end‐diastole indexed to body surface area; iRVFWd, right ventricular free wall thickness at end‐diastole indexed to body weight; iTAPSE, tricuspid annular plane systolic excursion indexed to body weight; Mean PG, mean pulmonary stenosis pressure gradient; OR, odds ratio; V_MaxAo_:V_MaxPV_, aorta‐to‐pulmonary artery maximum transvalvular velocity ratio; VTI_Ao_:VTI_PV_, aorta‐to‐pulmonary artery velocity time integral ratio; TR, tricuspid regurgitation severity.

The results of univariate and multivariate logistic regression analysis to identify echocardiographic variables associated with clinical severity of PS are presented in Table 3. Decreased iTAPSE and increased iRVEDA were independently associated with the presence of clinical signs, whereas decreased iFAC and increased TR severity were independently associated with the presence of R‐CHF (Table 4).

**TABLE 3:** Results of univariate and multivariate logistic regression analysis of the echocardiographic variables associated with the clinical severity of PS (presence of exercise intolerance, syncope and/or right‐sided congestive heart failure).

Variables	Univariable logistic regression		Multiple logistic regression	
	OR (95% CI)	*P* value	OR (95% CI)	*P* value
AV_a_:PV_a_		.18	‐	‐
Max PG	1.01 (1.00‐1.02)	.001	‐	.07
Mean PG	1.03 (1.01‐1.03)	.001	‐	‐
V_MaxAo_:V_MaxPV_		.20	‐	‐
VTI_Ao_:VTI_PV_	0.001 (0.001‐0.003)	.001	‐	.89
iPVA	0.013 (0.001‐0.21)	.002	‐	.85
iRAA	1.12 (1.04‐1.2)	.001	‐	.26
iRVEDA	1.18 (1.03‐1.35)	.01	**1.4 (1.08‐2.02)**	**.01**
iRVFWd	‐	.05	‐	‐
iTAPSE	0.52 (0.29‐0.91)	.02	**0.35 (0.15‐0.77)**	**.01**
iFAC	0.96 (0.93‐0.99)	.02	‐	.81
TR severity	2.5 (1.5‐4.3)	<.001	‐	.89

*Note*: The bold values represent the variables resulted statistically significant in the multivariable analysis.

Abbreviations: 95% CI, 95% confidence interval; AV_a_:PV_a_, aorta‐to‐pulmonary artery annulus ratio; iFAC, fractional area change indexed to body weight; iPVA, pulmonary valve area indexed to body surface area; iRAA, right atrial area indexed to body surface area; iRVEDA right ventricular area at end‐diastole indexed to body surface area; iRVFWd, right ventricular free wall thickness at end‐diastole indexed to body weight; iTAPSE, tricuspid annular plane systolic excursion indexed to body weight; Max PG, maximum transpulmonary pressure gradient; Mean PG. mean pulmonary stenosis pressure gradient; OR, odds ratio; V_MaxAo_:V_MaxPV_, aorta‐to‐pulmonary artery maximum transvalvular velocity ratio; VTI_Ao_:VTI_PV_, aorta‐to‐pulmonary artery velocity time integral ratio; TR severity, tricuspid regurgitation severity.

**TABLE 4:** Results of univariate and multivariate logistic regression analysis of echocardiographic variables associated with the presence of right‐sided congestive heart failure in dogs with PS.

Variables	Univariable logistic regression		Multiple logistic regression	
	OR (95% CI)	*P* value	OR (95% CI)	*P* value
AV_a_:PV_a_	5.6 (1.01‐31.7)	.05		.43
Max PG	1.01 (1.00‐1.02)	.03		.07
V_MaxAo_:V_MaxPV_	0.001 (0.001‐0.481)	.03		.72
VTI_Ao_:VTI_PV_	0.001 (0.001‐0.005)	.02		.83
iPVA	0.004 (0.001‐0.379)	.02		.18
iRAA	1.20 (1.09‐1.31)	<.001		.45
iRVEDA	1.25 (1.07‐1.46)	.004		.67
iRVFWd	19.2 (1.4‐262)	.03		.69
iTAPSE	0.42 (0.18‐0.99)	.05		.45
iFAC	0.92 (0.88‐0.96)	<.001	**0.93 (0.88‐0.99)**	**.02**
TR severity	8.2 (3.0‐22.4)	<.001	**6.68 (2.1‐20.3)**	**<.001**

*Note*: The bold values represent the variables resulted statistically significant in the multivariable analysis.

Abbreviations: 95% CI, 95% confidence interval; AV_a_:PV_a_, aorta‐to‐pulmonary artery annulus ratio; iFAC, fractional area change indexed to body weight; iPVA, pulmonary valve area indexed to body surface area; iRAA, right atrial area indexed to body surface area; iRVEDA right ventricular area at end‐diastole indexed to body surface area; iRVFWd, right ventricular free wall thickness at end‐diastole indexed to body weight; iTAPSE, tricuspid annular plane systolic excursion indexed to body weight; Max PG, maximum transpulmonary pressure gradient; OR, odds ratio; V_MaxAo_:V_MaxPV_, aorta‐to‐pulmonary artery maximum transvalvular velocity ratio; VTI_Ao_:VTI_PV_, aorta‐to‐pulmonary artery velocity time integral ratio; TR severity, tricuspid regurgitation severity.

Lastly, the results of the receiver operating curve analysis to calculate the cut‐offs for those variables that were significant in multivariate analysis are presented in Table 5. In particular, iRVEDA >8.6 cm^2^/m^2^ and iTAPSE <5.8 mm/kg^0.297^ were associated with clinical signs with a sensitivity of 71.4% (95%CI, 51.3‐86.8%) and 78.9% (95%CI, 54.4‐93.9%) and a specificity of 58.6% (95%CI, 44.9‐71.4%) and 48.7% (95%CI, 32.9‐64.9%), respectively. Moreover, a TR severity ≥2 was associated with the presence of R‐CHF with a sensitivity of 92.3% (95%CI, 64‐99.8%) and a specificity of 85.3% (95%CI, 75.3‐92.4%).

**TABLE 5:** Results of receiver operating curve analysis of variables associated with clinical signs (exercise intolerance, syncope and right congestive heart failure) and presence of right congestive heart failure.

	Cut‐off	AUC	*P* value	Se (%)	95% CI	Sp (%)	95% CI
Predictors of clinical signs							
iRVEDA (cm^2^/m^2^)	>8.6	.66	.001	71.4	51.3‐86.8	58.6	44.9‐71.4
iTAPSE (mm/kg^0.297^)	<5.8	.67	.01	78.9	54.4‐93.9	48.7	32.9‐64.9
Predictors of R‐CHF							
iFAC (%/kg^−0.097^)	≤52	.75	<.001	61.5	31.6‐86.1	84.9	74.6‐92.2
TR severity	≥2	.92	<.001	92.3	64‐99.8	85.3	75.3‐92.4

Abbreviations: 95% CI, 95% confidence interval; iFAC, fractional area change indexed to body weight; iRVEDA right ventricular area at end‐diastole indexed to body surface area; iTAPSE, tricuspid annular plane systolic excursion indexed to body weight; PS, pulmonary stenosis; R‐CHF, right sided congestive heart failure; Se, sensitivity; Sp, specificity; TR severity, tricuspid regurgitation.

## 4 DISCUSSION

We identified right heart echocardiographic variables associated with echocardiographic and clinical severity in dogs with PS and their cut‐offs for the presence of clinical signs of R‐CHF.

Regarding echocardiographic severity, increased iRVFWd and decreased VTI_Ao_:VTI_PV_ were independently associated with PS max PG > 80 mm Hg. In our study, consistent with a previous study,^8^ almost all dogs with PS PG >80 mm Hg had an increased iRVFWd (86% and 97%, respectively). In fact, in patients with PS, the RV responds to the increased afterload with myocyte hypertrophy leading to increased RV wall thickness.^8^ Therefore, it is not unexpected that iRVFWd is increased in the majority of dogs with a PS PG >80 mm Hg.

Similarly, the previous study found a mean value for VTI_Ao_:VTI_PV_ of 0.16 in a population of dogs with PS PG >80 mm Hg.^8^ The VTI_Ao_:VTI_PV_ ratio is a less flow dependent index and, as suggested by a previous study,^7^ might be a useful index to evaluate the severity of PS in dogs, especially in cases with high or low cardiac output.

Increased iRVEDA and decreased iTAPSE were independently associated with the presence of clinical signs. This observation is consistent with a previous study in dogs with PS that identified iTAPSE as the echocardiographic variable associated with the presence of clinical signs^8^ and, as previously suggested, should be included in the echocardiographic evaluation of dogs with PS to help predict clinical severity. However, in our study, the percentage of dogs with decreased iTAPSE was lower than in a previous study^8^ in both the overall canine population with PS (25% versus 81%) and in those with PS max PG >80 mm Hg (33% versus 86%). This difference can be explained by different study samples. Our investigation included a high proportion of dogs with TR (62.5%). Although the influence of TR on iTAPSE in dogs with PS has not been studied yet, similar to the other echocardiographic variables that evaluate RV systolic function (eg, iFAC),^24^, ^25^ it is known that iTAPSE is influenced by loading conditions in humans. Therefore, we cannot totally exclude that TR might have increased iTAPSE values in our population of dogs with PS, which might explain the difference between our study and the previous study.^8^ However, the maintenance of systolic function in humans and dogs could be explained by the fact that, in experimental studies, pressure overload in PS patients leads to cardiomyocyte hyperplasia rather than hypertrophy.^26^, ^27^ Moreover, our results are in agreement with a study in humans, that found that TAPSE values were within normal limits in the majority of PS patients.^28^ However, the value used to define decreased iTAPSE in previous studies originated from a population of adult dogs and not puppies.^20^ In human medicine, adults and children have different reference ranges of TAPSE and lower values are observed in children.^29^ In both our study and the previous study,^8^ the median age was approximately 1 year, and hence the cut‐off of TAPSE used to define RV systolic dysfunction might be inappropriate for these young dogs. Therefore, future studies are needed to define the reference range of TAPSE in puppies. Lastly, another reason that might explain the difference of TAPSE values between our study and the previous study^8^ might be different image acquisition, interoperator measurement variability and the multicenter nature of our study. In another study^19^ evaluating reference intervals and repeatability of right heart echocardiographic measurements, TAPSE showed a high coefficient of variation, with inter‐operator variability of 21%.

Notwithstanding, TAPSE might not be the best echocardiographic index to evaluate RV systolic function because it is highly influenced by loading conditions, by left ventricular systolic function (ventricular interdependence), and because it is an angle‐dependent parameter.^24^ In human medicine, it has been noted that strain and strain rate might be superior in evaluating RV systolic function, because they are angle‐independent, less load‐dependent and can detect systolic dysfunction in the early stage of disease both in congenital and acquired right heart diseases.^26^, ^28^, ^30^, ^31^ However, to our knowledge, no studies have assessed strain and strain rate in evaluating systolic function in dogs affected by PS and additional studies are needed to assess this aspect of the disease.

Ours is the first study that identified the echocardiographic variables associated with the presence of R‐CHF in dogs with PS. Identifying patients that may develop R‐CHF is extremely important because it is linked to cardiac‐related death and represents a negative prognostic factor.^4^ In fact, dogs with PS and R‐CHF have a life expectancy of approximately 12 months, which is significantly lower than in dogs without R‐CHF.^4^, ^15^ The only variables in our study that were independently associated with R‐CHF were iFAC and TR severity. The iFAC of the RV is another echocardiographic index that helps to evaluate RV systolic function. A recent study comparing echocardiography and magnetic resonance imaging to evaluate RV systolic function in healthy dogs indicated that FAC is better associated with magnetic resonance imaging parameters than TAPSE.^32^ This finding is not surprising because, as has been observed in humans, iFAC evaluates global RV systolic function (with the exception of RV outflow tract function), whereas iTAPSE assesses only longitudinal function of a single segment of the RV, excluding the contribution of other myocardial segments on RV performance.^25^ Therefore, consistent with previous studies in humans and dogs,^25^, ^32^, ^33^ iFAC could be considered a useful index for the evaluation of global RV systolic function in dogs with PS. Additional studies are needed to evaluate which is the better index to evaluate right ventricular systolic function in dogs with PS. However, in our study as in the previous study,^8^ iTAPSE was independently associated with clinical signs in dogs with PS and combining both of these echocardiographic indexes (iTAPSE and iFAC) might provide a more comprehensive evaluation of RV systolic function and clinical severity in dogs with PS.

In our study, an iTAPSE <5.8 mm/kg^0.297^ and iFAC <52%/kg^−0.097^ were associated with clinical signs and R‐CHF, respectively. However, these values are above the previously reported cut‐offs used to define RV systolic dysfunction (iTAPSE <4.8 mm/kg^0.297^ and iFAC <46.3%/kg^−0.097^).^19^, ^20^ This result may be due to the characteristics of dogs included in this series; TR in some cases might have enhanced RV preload by increasing these indexes and obscuring underling systolic dysfunction. Moreover, approximately half of the dogs were brachycephalic, with possibly different reference intervals for evaluation of RV systolic function. In fact, a study providing echocardiographic reference intervals in English Bulldogs indicated that left ventricular ejection fraction is higher in this breed than in the general population of dogs.^34^ Moreover, the study that provided the reference range of RV systolic function included 80 dogs, but excluded all brachycephalic breeds.^20^ Another possibility is that dogs with PS already start to develop clinical signs when RV systolic function indices are close to the lower reference interval, which might indicate an early stage of systolic dysfunction.

Tricuspid regurgitation severity, and in particular the presence of more than mild TR (TR ≥ 2), showed the highest accuracy in identifying dogs with R‐CHF in our study with a sensitivity of 92.3% and specificity of 85.3%. When the RV dilates in the late stage of the disease, it can stretch the tricuspid valve annulus giving rise to TR, which promotes right atrium enlargement and predisposes to development of R‐CHF. Therefore, the presence of TR, in the absence of tricuspid congenital abnormalities, might be considered an indicator of PS severity.^3^, ^5^ This result is consistent with 2 previous investigations that identified TR as a negative prognostic factor in dogs with PS.^3^, ^5^

Our study had some limitations. It was a cross‐sectional observational study, and our results should be confirmed by a longitudinal study. Moreover, the clinical signs of exercise intolerance were subjectively reported by owners, and a syncopal episode could have been missed by the owners in some cases. Our study included a high proportion of brachycephalic dogs that might have suffered from exercise intolerance because of their respiratory disease. Holter examination was not performed in dogs with syncope, and therefore it cannot be excluded that arrhythmias contributed to clinical signs, especially in Boxer and English Bulldogs that can be affected by arrhythmogenic RV cardiomyopathy.^35^, ^36^ However, the majority of dogs were young, making arrhythmogenic RV cardiomyopathy less likely.

One third of dogs in our study were treated with the atenolol, which can influence echocardiographic parameters. In particular, atenolol might have negatively affected RV systolic function^3^, ^6^ and decreased the PS PG, leading to a decrease in RV functional echocardiographic parameters, such as FAC and TAPSE, and to a misclassification of PS severity.^3^, ^6^ However, all dogs treated with atenolol had PS PG >80 mm Hg, and thus the influence of atenolol on classification of PS severity was marginal in our study. Moreover, as previously shown,^6^ less flow‐dependent parameters, such as iPVA, VTI_Ao_:VTI_PV_, V_MaxAo_:V_MaxPV_, are not greatly affected by beta‐blockers, and thus the use of atenolol may not have changed the results of these indices. However, the influence of atenolol on the different echocardiographic variables in our population was not evaluated in our study and we cannot exclude that this drug could have influenced the results of the observed variables, especially those assessing RV systolic function (ie, iTAPSE and iFAC).

In our study based only on PG of PS, 1 dog with afterload mismatch and R‐CHF was included in the group having a PS max PG ≤80 mm Hg, which mostly consisted of asymptomatic dogs. This finding reinforces the concept of using a multiparametric approach to stratify PS severity not only based on PG, but also by evaluation of right heart structure (ie, iRAA, iRVEDA), function (ie, FAC, TAPSE), and taking into consideration less‐flow dependent echocardiographic parameters (ie, iPVA, VTI_Ao_:VTI_PV_, V_MaxAo_:V_MaxPV_). Moreover, right atrial enlargement, ventricular enlargement, and systolic dysfunction were defined based on previous published cut‐offs derived from a population of healthy adult dogs.^18^, ^19^ In our study, almost 40% of dogs were <1 year of age, and it cannot be excluded that puppies have a different reference range for these variables compared to adult dogs. Moreover, 44% of the dogs in our study were brachycephalic. A previous study determined that, in particular, English Bulldogs have a different left‐sided cardiac chamber reference range for dimension, function, and geometry compared to the general dog population.^33^ It is possible that differences also are present for RV echocardiographic parameters.

In our study, the results of the association between echocardiographic variables and R‐CHF must be considered as preliminary based on the small sample size (only 11 dogs in R‐CHF). However, the association between R‐CHF and severity of TR was very strong and it is interesting that TR severity seems to be a main determinant of the presence of R‐CHF in both congenital and acquired right heart pressure‐overload diseases in dogs.^16^ Lastly, in our study TR severity was classified based only on a qualitative and semiquantitative assessment of the color and spectral Doppler signals. The echocardiographic evaluation of TR severity remains challenging both in human and veterinary medicine, and may present substantial limitations. In human medicine 2‐D and 3‐dimensional quantitative echocardiographic indices have been proposed in the evaluation of TR severity (eg, vena contracta, proximal isovelocity surface area, regurgitant volume calculation).^37^ However, to our knowledge, no studies in veterinary medicine evaluated these quantitative methods to assess TR severity and additional studies are needed to assess the feasibility, repeatability, and accuracy of these measurements in dogs.

## 5 CONCLUSION

Our study supports the use of different right heart echocardiographic variables to evaluate dogs affected by PS. In particular, decreased RV ventricular systolic function (ie, iTAPSE and FAC) and RV dilatation (ie., iRVEDA) are associated with the presence of clinical signs.

## CONFLICT OF INTEREST STATEMENT

Eric Zini serves as Associate Editor for the Journal of Veterinary Internal Medicine. He was not involved in review of this manuscript. No other authors declare a conflict of interest.

## OFF‐LABEL ANTIMICROBIAL DECLARATION

The authors declare no off‐label use of antimicrobials.

## INSTITUTIONAL ANIMAL CARE AND USE COMMITTEE (IACUC) OR OTHER APPROVAL DECLARATION

The authors declare no IACUC or other approval was needed.

## HUMAN ETHICS APPROVAL DECLARATION

The authors declare human ethics approval was not needed for this study.
